# Molecular tumor board: molecularly adjusted therapy upon identification and functional validation of a novel *ALK* resistance mutation in a case of lung adenocarcinoma

**DOI:** 10.1093/oncolo/oyae143

**Published:** 2024-07-03

**Authors:** Annette Arndt, Christian Neumann, Armin Riecke, Arthur Bauer, Matthias Müller, Manuela Wölfle-Guter, Michael Grunert, Hauke Busch, Axel Künstner, Nikolas von Bubnoff, Stephanie Fliedner, Dina Greinert, Jasmin Osius, Kumar Nagarathinam, Konrad Steinestel, Sivahari Prasad Gorantla, Niklas Gebauer, Hanno M Witte

**Affiliations:** Institute for Pathology and Molecular Pathology, Bundeswehrkrankenhaus Ulm, 89081 Ulm, Germany; Department of Hematology and Oncology, Bundeswehrkrankenhaus Ulm, 89081 Ulm, Germany; Department of Hematology and Oncology, Bundeswehrkrankenhaus Ulm, 89081 Ulm, Germany; Department of Hematology and Oncology, Bundeswehrkrankenhaus Ulm, 89081 Ulm, Germany; Department of Hematology and Oncology, Bundeswehrkrankenhaus Ulm, 89081 Ulm, Germany; Internistische Praxisgemeinschaft Ehingen, 89584 Ehingen, Germany; Department of Nuclear Medicine, Bundeswehrkrankenhaus Ulm, 89081 Ulm, Germany; Department of Nuclear Medicine, University Hospital Ulm, 89081 Ulm, Germany; Medical Systems Biology Group, University of Lübeck, 23538 Lübeck, Germany; Institute for Cardiogenetics, University of Lübeck, 23538 Lübeck, Germany; University Cancer Center Schleswig-Holstein, University Hospital of Schleswig-Holstein, 23538 Lübeck, Germany; Medical Systems Biology Group, University of Lübeck, 23538 Lübeck, Germany; Institute for Cardiogenetics, University of Lübeck, 23538 Lübeck, Germany; University Cancer Center Schleswig-Holstein, University Hospital of Schleswig-Holstein, 23538 Lübeck, Germany; University Cancer Center Schleswig-Holstein, University Hospital of Schleswig-Holstein, 23538 Lübeck, Germany; Department of Hematology and Oncology, University Hospital of Schleswig-Holstein, 23538 Lübeck, Germany; Institute for Cardiogenetics, University of Lübeck, 23538 Lübeck, Germany; University Cancer Center Schleswig-Holstein, University Hospital of Schleswig-Holstein, 23538 Lübeck, Germany; University Cancer Center Schleswig-Holstein, University Hospital of Schleswig-Holstein, 23538 Lübeck, Germany; Department of Hematology and Oncology, University Hospital of Schleswig-Holstein, 23538 Lübeck, Germany; University Cancer Center Schleswig-Holstein, University Hospital of Schleswig-Holstein, 23538 Lübeck, Germany; Department of Hematology and Oncology, University Hospital of Schleswig-Holstein, 23538 Lübeck, Germany; Department of Biochemistry, University of Lübeck, 23538 Lübeck, Germany; Institute for Pathology and Molecular Pathology, Bundeswehrkrankenhaus Ulm, 89081 Ulm, Germany; University Cancer Center Schleswig-Holstein, University Hospital of Schleswig-Holstein, 23538 Lübeck, Germany; Department of Hematology and Oncology, University Hospital of Schleswig-Holstein, 23538 Lübeck, Germany; University Cancer Center Schleswig-Holstein, University Hospital of Schleswig-Holstein, 23538 Lübeck, Germany; Department of Hematology and Oncology, University Hospital of Schleswig-Holstein, 23538 Lübeck, Germany; Institute for Pathology and Molecular Pathology, Bundeswehrkrankenhaus Ulm, 89081 Ulm, Germany; Department of Hematology and Oncology, Bundeswehrkrankenhaus Ulm, 89081 Ulm, Germany; University Cancer Center Schleswig-Holstein, University Hospital of Schleswig-Holstein, 23538 Lübeck, Germany; Department of Hematology and Oncology, University Hospital of Schleswig-Holstein, 23538 Lübeck, Germany

**Keywords:** ALK translocations, molecular profiling, functional validation, NSCLC, ALK inhibitors

## Abstract

We report a case of a long-term surviving patient with *EML4/ALK* translocated non–small cell adenocarcinoma of the lung in UICC8 stage IVA. During recurrence under continuous crizotinib therapy, a hitherto insufficiently characterized missense mutation in the *ALK* gene (Arg1181His) was identified through targeted sequencing. The aforementioned *EML4/ALK* translocation could still be detected in this situation. Employing a 3D reconstruction of the ALK tertiary structure, considering its interaction with various ALK inhibitors at the molecular binding site, our analysis indicated the presence of a mutation associated with crizotinib resistance. To validate the biological relevance of this previously unknown mutation, we carried out an in vitro validation approach in cell culture in addition to the molecular diagnostics accompanied by the molecular tumor board. The tumor scenario was mimicked through retroviral transfection. Our comparative in vitro treatment regimen paired with the clinical trajectory of the patient, corroborated our initial clinical and biochemical suspicions. Our approach demonstrates preclinical, in silico, and clinical evidence of a novel crizotinib resistance mutation in ALK as well as sensitivity toward brigatinib and potentially lorlatinib. In future cases, this procedure represents an important contribution to functional diagnostics in the context of molecular tumor boards.

## Teaser

This article reports the case of a long-term surviving patient with EML4/ALK translocated non–small cell adenocarcinoma of the lung in UICC8 stage IVA. In progression under continuous crizotinib therapy, a hitherto insufficiently characterized missense mutation in the ALK gene (Arg1181His) was identified using targeted sequencing and then validated as the mechanism of resistance in a molecularly exhaustive approach.

## Graphical Abstract

**Graphical Abstract:**
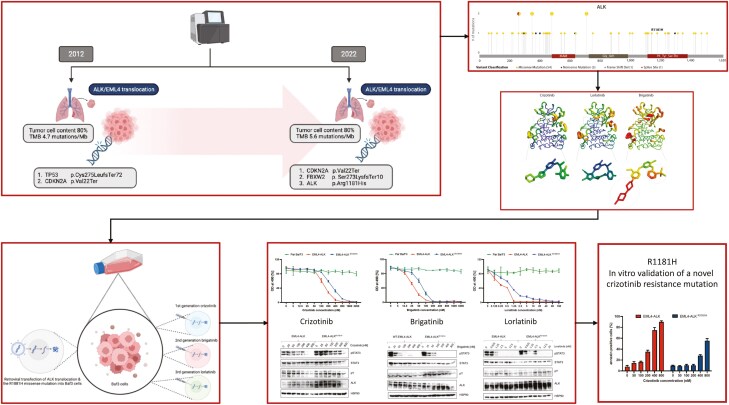

**Key points**

- This article describes for the first time, to the best of the authors’ knowledge, the association between an acquired ALK R1181H mutation and partial resistance toward crizotinib in EML4/ALK translocated adenocarcinoma of the lung.
- The approach presented here including in silico and in vitro experiments can be used as an example for the functional validation of newly emerged potential resistance mutations.
- The inclusion of functional validations of specific genetic features serves as an additional component for treatment guidance and the increase of efficacy in molecularly stratified therapies.

## Introduction

Translocations involving the anaplastic lymphoma kinase (*ALK*) represent common oncogenic events in 4%-5% of patients with non–small cell lung cancer (NSCLC).^1^ Echinoderm microtubule-associated protein-like 4 (*EML4*) acts as a translocation partner and is involved most frequently in *ALK* translocations leading to increased activity of the *ALK* tyrosine kinase.^2^ Activating *ALK* translocations promote proliferation and tumor growth. The occurrence of *ALK* translocations is associated with a non-/never smoking status, younger age, and histology of adenocarcinoma subtype.^3^ In metastatic disease, *ALK* inhibitors are used as the standard of care in the frontline setting.^4^ Their introduction significantly improved patient outcomes across the past decade.^4^ Several ALK inhibitors with increasing efficacy from generation to generation are approved for first-line therapy and in the relapsed/refractory setting if an *ALK* translocation is detectable.^4,5^ However, the efficacy of ALK inhibitor treatment is of limited duration due to primary or acquired drug resistance.^6^ In the era of comprehensive molecular profiling, the body of evidence on resistance mechanisms in cancers harboring *ALK* translocations is growing.^7^

## Patient story

**Figure 1.**
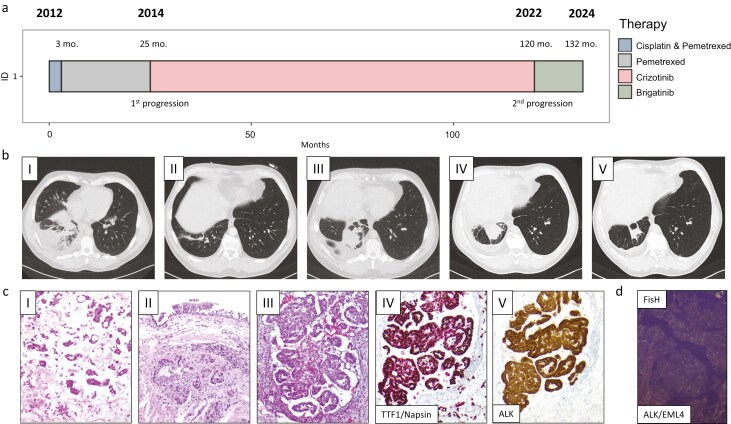
Clinical, therapeutic, CT graphic, and histopathologic sequence of the disease. (a) Swimmer plot, which illustrates the clinical course and the sequence of therapies administered. (b) 47-year-old man with NSCLC cTxpN3 pM1a (ple). CT scans in initial staging and follow-up. (I) Initial CT and PET imaging (not shown) after malignancy confirmation in pleural biopsy revealed advanced-stage disease with primary tumor localization in the right lower lobe and adjacent atelectasis. (II) After 3 chemotherapy cycles of cisplatin/pemetrexed and pemetrexed maintenance treatment, a partial response (PR) was achieved. (III) CT scan after 22 months showed progressive disease (PD) with a significant increase in size; second-line crizotinib was initiated. (IV) Progression 120 months after initial diagnosis and 96 months after crizotinib initiation. (V) First CT scan evaluation 3 months after TKI switch to brigatinib revealed partial remission and current CT shows persistent stable disease up to 11 months after change in therapy (not shown). (c) Representative microphotographs of tumor samples from 2012 (I), 2014 (II), and 2022 (III) including immunohistochemical stainings for TTF-1/Napsin (IV; double-staining) and ALK (V; clone D5F3). Abbreviations: TTF-1, thyroid transcription factor 1; ALK, anaplastic lymphoma kinase. Scale bar = 100 µm. (d) The detection of the *EML4/ALK* fusion in FISH diagnostics.

A 47-year-old never-smoker male patient presented with irritable cough, progressive shortness of breath, and deep vein thrombosis in the left lower leg at initial diagnosis. The patient was a radar technician for the German Air Force. The initial computer tomography (CT) scan of the chest revealed a pulmonary mass in the right lower lobe measuring 55 × 44 mm. Staging diagnostics including positron-emission-tomography (PET)-CT and cytologic studies confirmed extensive lymphonodal involvement affecting the left supraclavicular, axillar as well as infracarinal, hilar, paratracheal, aortopulmonary, and mediastinal region with right-sided malignant pleural effusion. Histopathological workup led to the diagnosis of moderately differentiated non–small cell adenocarcinoma of the lung (NSCLC) UICC8-stage IVA (cTx pN3 pM1a; Figure 1c). Fluorescence in-situ hybridization (FisH) identified *EML4/ALK* rearrangement acting as the potential driver of the disease. Moreover, molecular analysis of the *EGFR-*gene locus detected a wild-type constellation. Based on the guidelines valid at the time, no further possible driver mutations were initially investigated.

In 2012, previous to the era of immunotherapy and the widespread implementation of targeted treatment strategies in NSCLC, systemic chemotherapy with cisplatin and pemetrexed represented the standard of care regardless of the mutational status in adenocarcinoma subtypes. Combination chemotherapy was stopped after 3 cycles due to severe ototoxicity followed by 22 months of pemetrexed maintenance until tumor progression (Figure 1a, 1b). Histopathological reevaluation confirmed the previously identified *ALK* translocation (Supplementary Figure S1). Consecutively, second-line treatment with crizotinib, a then-novel ALK inhibitor, was initiated 24 months after initial diagnosis. Crizotinib treatment resulted in long-term stable disease lasting for 95 months. Follow-up investigations during this period revealed persistent pericardial and right-sided pleural effusion, stable tumor mass in the right lower lobe and axillary as well as mediastinal lymph node involvement. In line with the increased risk for thrombogenesis associated with *ALK* translocations, the patient developed deep vein thrombosis of the right subclavian vein (7 months) with consecutive post-thrombotic syndrome of the right arm.^3^ A right-sided mild pneumothorax (58 months) and a non-necrotizing fasciitis of the left arm (99 months) complicated the course of the disease within the follow-up period.

Ten years after initial diagnosis, a follow-up CT scan of the chest demonstrated progression of the primary site in the right lower lobe (measuring 45 × 39 mm in transversal extension), 2 new suspicious lung nodules as well as an enhancement in the right-sided pleural effusion (Figure 1b). However, in keeping with the nature of a partial resistance mutation, the kinetics of progression were only moderate. Nevertheless, RECIST criteria for progression were met. Subsequent bronchoscopy was performed and molecular profiling identified an acquired potential ALK inhibitor resistance mutation (ALK protein: p.Arg1181His) without further evidence of other resistance mechanisms. To better understand the biology of the *ALK* missense mutation detected in our patient, an in vitro cell culture assay was added to functionally validate the mutation and to guide current as well as future treatment in conjunction with biochemical 3D-model calculations.

## Molecular Tumor Board

### Genotyping results and interpretation of the molecular results

**Figure 2.**
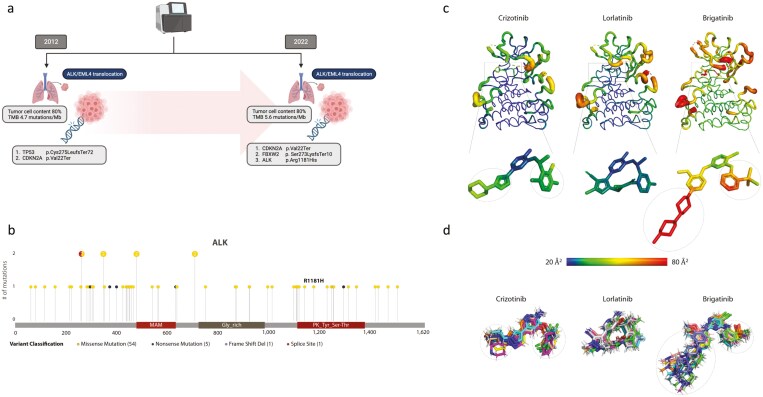
Overview of the results from molecular diagnostics. (a) Comparative ultra-deep panel sequencing of tissue material from initial diagnosis (2012) and recurrence after 8 years of crizotinib therapy (2022). Clonal evolution of the *CDKN2A*-bearing clone can be deduced. The loss of the *TP53* alteration in the more recent tissue sample is worth mentioning. A missense mutation in the *ALK* gene leading to a p.Arg1181His replacement was newly detected in 2022. (b) The Lollipop plot illustrates all resistance mutations in the ALK gene segments after extensive literature research. The p.Arg1181His mutation detected in the patient’s tissue sample is reported for the first time in NSCLC. (c, d) The B-factor of ALK-ligand complexes. (c) Putty representation of the ALK-ligand complex highlighting high to low flexible regions of the molecule color-coded in rainbow (low b-factor in blue, high b-factor in red). The ligands are also color-coded based on the rigid and flexible parts within the molecule (gray circle). (d) The ligand molecule from the trajectories highlighted with flexible groups (gray circle) over 100 ns of MD simulations.

We conducted comprehensive, ultra-deep high-throughput DNA next generation sequencing from novel tissue samples and initial diagnosis (primary tumor site) employing the TruSight Oncology 500 (TSO500) panel (median coverage 458x) on an Illumina NextSeqDX550 platform. An additional DNA sequencing approach confirmed *EML4* as a translocation partner of *ALK*. More specifically, the *EML4-ALK* variant E6;A19 could be identified (Supplementary Figure S1). However, the primary intention for DNA-based molecular reassessment was to identify characteristic mutations associated with ALK inhibitor resistance and, to potentially guide the further treatment strategy. Apart from the previously known *ALK* translocation, molecular diagnostics identified pathogenic variants in *CDKN2A* and *FBXW2* as well as an acquired single nucleotide variant (SNV) in the *ALK* gene (c.3542G > A, p.Arg.1181His; variant allele frequency 37.55%; coverage 1337x; Figure 2a). This SNV may potentially represent a resistance mutation as the locus of the present mutation is frequently involved in known mechanisms of TKI resistance (eg, p.Leu1196Met, p.Cys1156Tyr or p.Leu1152Arg; Figure 2b). To the best of our knowledge, this variant has so far not been reported in NSCLC (Supplementary Methods).

### Functional and clinical significance of the R1181H ALK mutation

To further evaluate the functional impact of the p.Arg.1181His missense mutation, we calculated biochemical 3D models of the resulting ALK molecule tertiary structure predicting the binding capacity in the context of molecular dynamics (MD) simulations of several ALK inhibitors (first generation: crizotinib; second generation: brigatinib; third generation: lorlatinib) in this case. Our in silico experiments (Supplementary Methods) suspected a potential responsiveness toward brigatinib and lorlatinib compared to crizotinib due to a more flexible beta-layer sheet which facilitates binding opportunities for the next generation ALK inhibitors (Figure 2c; Supplementary Table S1 and Figure S2). In addition, we calculated the binding energy with the different ALK inhibitors. These calculations suggest an increase in binding energy for lorlatinib in interaction with the mutated ALK structure (Supplementary Table S2 and Figure S2).

**Figure 3.**
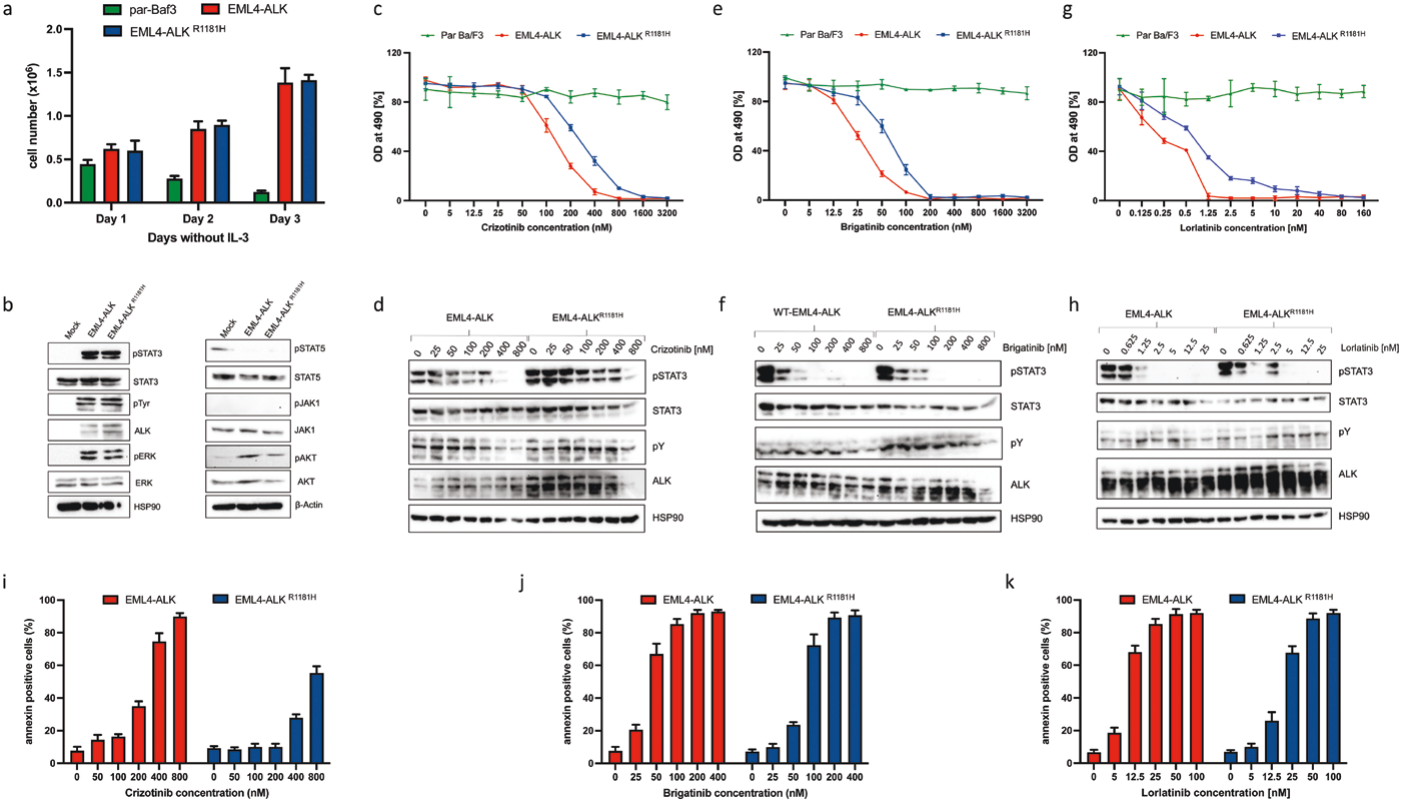
EML4/ALK^R1181H^ variant confers partial resistance to ALK-ATP competitive inhibitors compared to non-mutated EML4/ALK based on functional validation studies in vitro. (a) Absolute cell numbers over time were measured in the absence of IL-3 by trypan blue exclusion (*n* = 3). Data are shown as mean ±  SD. (b) Immunoblot analysis of serum-starved Ba/F3 cells expressing EML4/ALK or EML4/ALK^R1181H^ with indicated antibodies. (c) MTS (3-(4,5-dimethylthiazol-2-yl)-2,5-diphenyltetrazolium bromide)-based cell proliferation analysis of Ba/F3 cells expressing EML4/ALK and EML4/ALK^R1181H^ cultured with indicated concentrations of crizotinib for 48 hours. Data are shown as mean ± SD (*n* = 3). (d) Immunoblot analysis of Ba/F3 cells expressing EML4/ALK and EML4/ALK^R1181H^ cultured with indicated concentrations of crizotinib for 4 hours. A representative image of 2 independent experiments is shown (*n* = 2). (e) MTS-based cell proliferation analysis of Ba/F3 cells expressing EML4/ALK and EML4/ALK^R1181H^ with indicated concentrations of brigatinib for 48 hours. Data are shown as mean ± SD (*n* = 3). (f) Immunoblot analysis of Ba/F3 cells expressing EML4/ALK and EML4/ALK^R1181H^ cultured with indicated concentrations of brigatinib for 4 hours. A representative image of 2 independent experiments is shown (*n* = 2). (g) MTS-based cell proliferation analysis of Ba/F3 cells expressing EML4/ALK and EML4/ALK^R1181H^ with indicated concentrations of lorlatinib for 48 hours. Data are shown as mean ± SD (*n* = 3). (h) Immunoblot analysis of Ba/F3 cells expressing EML4/ALK and EML4/ALK^R1181H^ cultured with indicated concentrations of lorlatinib for 4 hours. (i) Annexin-V staining was performed on Ba/F3 cells expressing EML4/ALK and EML4/ALK^R1181H^ cultured with indicated concentrations of crizotinib for 48 hours. Data are shown as mean ± SD (*n* = 3). (j) Annexin-V staining was performed on Ba/F3 cells expressing EML4/ALK and EML4/ALK^R1181H^ cultured with indicated concentrations of brigatinib for 48 hours. Data are shown as mean ± SD (*n* = 3). (k) Annexin-V staining was performed on Ba/F3 cells expressing EML4/ALK and EML4/ALK^R1181H^ cultured with indicated s of lorlatinib for 48hrs. Data are shown as mean ± SD (*n* = 3). Abbreviation: OD, optical density.

This biophysical hypothesis had to be functionally validated in vitro. We therefore cloned the *EML4/ALK* translocation (EML4/ALK variant) as well as the p.Arg1181His missense mutation (EML4/ALK^R1181H^ variant) into an NSCLC cell line (BaF3) via the retroviral vector PIG (plasmid-IRES-EGFP-Puromycin). Next, we demonstrated that the EML4/ALK^R1181H^ variant is equally oncogenic as the EML4/ALK variant (Figure 3a; Supplementary Figure S3). Afterward, we found that *STAT3, ERK,* and *AKT* are strongly activated in both EML4/ALK cells and the EML4/ALK^R1181H^ variant expressing cells, suggesting the crucial role of STAT3 which is independent of JAK family kinase, MAP kinase and PI3K pathways activation in ALK-transfected cells (Figure 3b). Analysis of resistance toward different ALK inhibitors showed that the cellular IC50 value in the EML4/ALK^R1181H^ variant was 3 times higher (~300 nM) compared to the wild-type EML4/ALK (~105 nM) variant in the setting of in vitro treatment with crizotinib (Figures 3c, 3d; Supplementary Table S3). Consistent with the cell proliferation data, biochemical analysis of ALK and STAT3 phosphorylation data also show that both variants EML4/ALK and EML4/ALK^R1181H^ are inhibited at nanomolar concentrations of lorlatinib and brigatinib compared to crizotinib, suggesting that second and third generation ALK inhibitors are more potent toward the variants which drive partial resistance toward crizotinib (Figures 3e-3h; Supplementary Figure S4). Finally, we also analyzed the induction of apoptosis after ALK inhibitor treatment in these cells and found that the EML4/ALK^R1181H^ variant is partially resistant toward crizotinib compared to brigatinib and lorlatinib (Figures 3i-3k; Supplementary Figure S5 and Table S4). The in vitro test series is described in more detail in Supplementary Material section. In conclusion, we were able to validate our hypothesis of a new *ALK* resistance mutation that confers resistance toward crizotinib, but is still sensitive toward brigatinib as well as lorlatinib in vitro.

## Molecular Tumor Board recommendation

Upon relapse in 2022, we introduced our patient to the Molecular Tumor Board (MTB) of the University Cancer Center Schleswig-Holstein. In this context, MTB recommended a TKI switch to brigatinib which was based on the publication by Kim et al^8^ and current guidelines. Unfortunately, due to an insufficient quantity of tissue material, additional transcriptomic studies were not feasible. We will obtain a new biopsy to carry out extended molecular profiling in case of acquired brigatinib resistance.

## Patient update

Follow-up CT scans on brigatinib treatment demonstrated an ongoing PR per RECIST criteria 12 months after TKI switch (130 months after initial diagnosis). Brigatinib is well tolerated. The disease remains stable and the patient continues on brigatinib. In the event of progression, we would again obtain tissue material and quickly test for the presence of novel *ALK* mutations in order to analyze resistance mechanisms in the manner described above and to check the possibility of further TKI switch to lorlatinib.

## Discussion

The present manuscript reports on an acquired *ALK* resistance mutation in an *EML4/ALK*-rearranged non–small cell adenocarcinoma of the lung. The functionality of this missense mutation (p.Arg1181His) was validated in an individualized in vitro cell culture approach. The hypothesis that this mutation served as a mechanism of resistance toward first generation ALK inhibitor treatment with crizotinib was supported by 3D models calculating the spatial drug-molecule interaction. Ultimately, our predictions were confirmed by the clinical presentation upon 12 months follow-up CT scan evaluations resulting in satisfactory response on second-generation ALK inhibitor treatment with brigatinib and additionally, they are in line with our functional characterization of the mutation. The MTB monitored the present case of brigatinib treatment from initiation onwards.

The p.Arg1181His mutation has only been reported once in an NSCLC case at initial diagnosis in which the authors noticed partial sensitivity toward alectinib as well as once in cervical carcinoma.^9,10^ Here, the mutation was suspected to increase the function of the involved protein kinase (amino acid sequence from 1110 to 1384). Consecutively, this might not only lead to resistance toward crizotinib treatment but also augments the role of *ALK* in carcinogenesis and disease progression.^11^

In recent years, there is growing evidence regarding mechanisms of resistance toward targeted therapies, especially in the spectrum of ALK inhibitor treatment as this modularity potentially affects several malignancies in the era of precision oncology.^12^ Resistance can be subdivided into primary and acquired resistance. Primary resistance is associated with a lack of TKI treatment response immediately after initiation. Acquired resistance to ALK inhibitors on the other hand can be categorized into 2 subgroups, on-target and off-target mechanisms.^7^ To date, 96 decisive on-target resistance mutations have been described in NSCLC, the entity in which ALK inhibitors are administered most frequently in clinical routine.^7^ Alterations in bypass signaling pathways indirectly affecting the *ALK* gene functionality represent the essential mechanism for the development of off-target resistance. SNVs or insertions/deletions (indels) in *EGFR*, *BRAF*, *KRAS*, *TP53*, *PIK3CA*, *MEK*, *IGF-1R*, *SRC*, or *HER*, amplifications in *KIT* or *MET* and *RET* fusions were previously described as potential off-target resistance alterations.^7^ Moreover, the transformation into small cell lung cancer (SCLC) or epithelial-to-mesenchymal transition which leads to the loss of polarity and intercellular connections of epithelial cells and enhances migration and invasion of tumor cells represent additional off-target resistance mechanisms.^13^

Knowledge regarding frequent on-target resistance mutations across the *ALK* gene is steadily increasing.^6^ In their review article, Pan et al^7^ summarized preclinical and clinical data on the tumor-biologic effect (sensitivity versus resistance) of several on-target *ALK* mutations concerning approved ALK inhibitors. However, nothing was known about the functional implications of the detected p.Arg1181His mutation in NSCLC. Thus, 3D-model calculations and the in vitro validation were able to underline the relevance of this mutation expanding the field of on-target *ALK* resistance mutations which is of clinical importance.

Further advancements for the reliable detection of genomic alterations leading to ALK inhibitor resistance from the peripheral blood via liquid biopsies analyzing cell-free DNA fragments (cfDNA)/circulating tumor DNA (ctDNA) are urgently warranted for optimal treatment guidance. To date, the essential critical aspects regarding the implementation of liquid biopsies in routine clinical use address the sensitivity and lack of technological standardization between laboratories as well as pending results from prospective studies.^14^ In the era of precision oncology, we believe that liquid biopsies and functional validation studies to anticipate the therapeutic efficacy as performed here will enter the currently developing field of diagnostics that can dynamically monitor and guide cancer treatments.

Currently, clinical experience and available molecular insights support the sequential application of ALK inhibitors in case of progression and afterward adjusting the treatment strategy with another ALK inhibitor since the development of on-target resistance mutations seems to underlie a hierarchical process.^15^ Some data suggest that a combination of ALK inhibitors with other targeted therapeutics offers promising possibilities to overcome resistance.^16^

In summary, we were able to functionally and clinically validate our hypothesis that brigatinib can overcome resistance toward first generation ALK inhibitors in the presence of an acquired p.Arg1181His mutation.^17^ Additionally, brigatinib and lorlatinib outperformed first generation ALK inhibitor crizotinib in the modified *EML4/ALK*-rearranged BaF3 cell line harboring the transfected p.Arg1181His mutation.

## Supplementary Material

oyae143_suppl_Supplementary_Material

oyae143_suppl_Supplementary_Table_S4

## Data Availability

The data underlying this article will be shared on reasonable request to the corresponding author.

## References

[1] Aguado de la Rosa C , Cruz Castellanos P, Lázaro-Quintela M, et al. Identification of ALK-positive patients with advanced NSCLC and real-world clinical experience with crizotinib in Spain (IDEALK study). Lung Cancer. 2022;173:83-93. 10.1016/j.lungcan.2022.09.010 36162227

[2] Du X , Shao Y, Qin HF, Tai YH, Gao HJ. ALK-rearrangement in non-small-cell lung cancer (NSCLC). Thorac Cancer. 2018;9(4):423-430. 10.1111/1759-7714.12613 29488330 PMC5879058

[3] Shaw AT , Yeap BY, Mino-Kenudson M, et al. Clinical features and outcome of patients with non-small-cell lung cancer who harbor EML4-ALK. J Clin Oncol. 2009;27(26):4247-4253. 10.1200/JCO.2009.22.6993 19667264 PMC2744268

[4] McCusker MG , Russo A, Scilla KA, Mehra R, Rolfo C. How I treat ALK-positive non-small cell lung cancer. ESMO Open. 2019;4(Suppl 2):e000524. 10.1136/esmoopen-2019-000524 31423342 PMC6677959

[5] Camidge DR , Kim HR, Ahn MJ, et al. Brigatinib versus crizotinib in ALK-positive non-small-cell lung cancer. N Engl J Med. 2018;379(21):2027-2039. 10.1056/NEJMoa1810171 30280657

[6] Koopman B , Groen HJM, Schuuring E, et al. Actionability of on-target ALK resistance mutations in patients with non-small cell lung cancer: local experience and review of the literature. Clin Lung Cancer. 2022;23(2):e104-e115. 10.1016/j.cllc.2021.06.011 34325996

[7] Pan Y , Deng C, Qiu Z, Cao C, Wu F. The resistance mechanisms and treatment strategies for ALK-rearranged non-small cell lung cancer. Front Oncol. 2021;11:713530. 10.3389/fonc.2021.713530 34660278 PMC8517331

[8] Kim DW , Tiseo M, Ahn MJ, et al. Brigatinib in patients with crizotinib-refractory anaplastic lymphoma kinase-positive non-small-cell lung cancer: a randomized, multicenter phase II trial. J Clin Oncol. 2017;35(22):2490-2498. 10.1200/JCO.2016.71.5904 28475456

[9] Consortium ITP-CAWG. Pan-cancer analysis of whole genomes. Nature. 2020;578(7793):82-93. 32025007 10.1038/s41586-020-1969-6 PMC7025898

[10] Tian X , Liao Q, Yang Q, et al. A novel alectinib-sensitive CTNND1-ALK fusion in a lung adenocarcinoma patient: a case report. Invest New Drugs. 2022;40(4):850-853. 10.1007/s10637-022-01245-3 35441911

[11] Della Corte CM , Viscardi G, Di Liello R, et al. Role and targeting of anaplastic lymphoma kinase in cancer. Mol Cancer. 2018;17(1):30. 10.1186/s12943-018-0776-2 29455642 PMC5817803

[12] Takeyasu Y , Okuma HS, Kojima Y, et al. Impact of ALK inhibitors in patients with *ALK*-Rearranged Nonlung Solid Tumors. JCO Precis Oncol. 2021;5:PO.20.00383. 10.1200/PO.20.00383 34036223 PMC8140781

[13] Dimou A , Lo YC, Merrell KW, Halling KC, Mansfield AS. Small cell transformation in a patient with *RET *Fusion-Positive Lung Adenocarcinoma on Pralsetinib. JCO Precis Oncol. 2022;6:e2200478. 10.1200/PO.22.00478 36542817

[14] Pailler E , Oulhen M, Borget I, et al. Circulating tumor cells with aberrant *ALK* Copy Number Predict Progression-Free Survival during Crizotinib Treatment in *ALK*-Rearranged Non-Small Cell Lung Cancer Patients. Cancer Res. 2017;77(9):2222-2230. 10.1158/0008-5472.CAN-16-3072 28461563

[15] Schneider JL , Lin JJ, Shaw AT. ALK-positive lung cancer: a moving target. Nat Cancer. 2023;4(3):330-343. 10.1038/s43018-023-00515-0 36797503 PMC10754274

[16] Cognigni V , Pecci F, Lupi A, et al. The landscape of ALK-rearranged non-small cell lung cancer: a comprehensive review of clinicopathologic, genomic characteristics, and therapeutic perspectives. Cancers (Basel). 2022;14(19):4765. 10.3390/cancers14194765 36230686 PMC9563286

[17] Okada K , Araki M, Sakashita T, et al. Prediction of ALK mutations mediating ALK-TKIs resistance and drug re-purposing to overcome the resistance. EBioMedicine. 2019;41:105-119. 10.1016/j.ebiom.2019.01.019 30662002 PMC6441848

