# Supplementary material for: Molecular tumor board: molecularly adjusted therapy upon identification and functional validation of a novel ALK resistance mutation in a case of lung adenocarcinoma
Source: Oncologist. 2024 Jul 3;30(1):oyae143. doi: 10.1093/oncolo/oyae143 (PMC11783293; doi:10.1093/oncolo/oyae143)
Supplement: oyae143_suppl_Supplementary_Material [file oyae143_suppl_supplementary_material.docx]

**Title: Molecular tumor board: Molecularly adjusted therapy upon identification and functional validation of a novel *ALK* resistance mutation in a case of lung adenocarcinoma**

Authors: Annette Arndt^1*^, Christian Neumann^2*^, Armin Riecke^2*^, Arthur Bauer^2^, Matthias Müller^2^, Manuela Wölfle-Guter^3^, Michael Grunert^4,5^, Hauke Busch^6,7,8^, Axel Künstner^6,7,8^, Nikolas von Bubnoff^8,9^, Stephanie Fliedner^7,8^, Dina Greinert^8,9^, Jasmin Osius^8,9^, Kumar Nagarathinam^10^, Konrad Steinestel^1^, Sivahari Prasad Gorantla^8,9#^, Niklas Gebauer^8,9#^, Hanno Witte^1,2,8,9#^

^* contributed equally^

^# shared senior authorship^

**Affiliations:**

^1^ Institute for Pathology and Molecular Pathology, Bundeswehrkrankenhaus Ulm, Oberer Eselsberg 40, 89081 Ulm, Germany

^2^ Department of Hematology and Oncology, Bundeswehrkrankenhaus Ulm, Oberer Eselsberg 40, 89081 Ulm, Germany

^3^ Internistische Praxisgemeinschaft Ehingen, Hopfenhausstraße 2, 89584 Ehingen, Germany

^4^ Department of Nuclear Medicine, Bundeswehrkrankenhaus Ulm, Oberer Eselsberg 40, 89081 Ulm, Germany

^5^ Department of Nuclear Medicine, University Hospital Ulm, Albert-Einstein-Allee 23, 89081 Ulm, Germany

^6^ Medical Systems Biology Group, University of Lübeck, Ratzeburger Allee 160, 23538 Lübeck, Germany

^7^ Institute for Cardiogenetics, University of Lübeck, Ratzeburger Allee 160, 23538 Lübeck, Germany

^8^ University Cancer Center Schleswig-Holstein, University Hospital of Schleswig- Holstein, Campus Lübeck, Ratzeburger Allee 160, 23538 Lübeck, Germany

^9^ Department of Hematology and Oncology, University Hospital of Schleswig-Holstein, Campus Lübeck, Ratzeburger Allee 160, 23538 Lübeck, Germany

^10^ Department of Biochemistry, University of Lübeck, Ratzeburger Allee 160, 23538 Lübeck, Germany

**Supplementary Material**

**Materials and Methods**

**Clinicopathological characteristics**

First, the case history was worked up chronologically. Clinical, laboratory and imaging characteristics were collected from the original electronic patient file. Histopathologic diagnostics were performed at the Institute of Pathology and Molecular Pathology, Bundeswehrkrankenhaus Ulm. This included conventional microscopy, immunohistochemistry and genomic profiling (see below).

**Molecular diagnostics**

For sequencing analysis, DNA and RNA were isolated from tumor tissue using the Maxwell®CSC DNA FFPE and Maxwell®CSC RNA FFPE kits on a Maxwell®CSC instrument according to the manufacturer´s protocols (Promega, Madison, WI, USA). Subsequently, the concentration of nucleic acids was determined fluorometrically (Qubit4 fluorometer; Thermo Fisher Scientific, Waltham, MA, USA) and quality control of genomic DNA as well as total RNA was tested by capillary gel electrophoresis (Fragment Analyzer System; Santa Clara, CA, USA). For DNA- and RNA-based targeted NGS, the TruSight Oncology 500 kit was used (San Diego, CA, USA). Therefore, 100ng of genomic DNA was fragmented by ultrasonication (ME 220 focused ultrasonicator, Brighton, UK), and 40ng of total RNA was converted into cDNA. The cDNA synthesis, together with RNA- and DNA-based library preparation was done exactly according to the manufacturer´s recommendations. Quantity and quality assessment as well as normalization and pooling of prepared libraries was also performed according to the TruSight Oncology 500 protocol. Next-generation sequencing was conducted loading 1pm of pooled libraries on the NextSeq550Dx sequencer machine (San Diego, CA, USA).

**Molecular dynamics simulations on ALK wildtype and mutant R1181H with ligand-complex or apo-form**

Molecular dynamics (MD) were performed on the PDB models 2YFX (crizotinib), 5A9U (lorlatinib) and 6MX8 (brigatinib) with the missing loops modelled using Modeller(1). The best models with the highest zDOPE score were selected as the initial coordinates for simulations as wildtype (WT) and the residue ARG1181 was mutated to HIS in the case of the mutant. Simulations on the WT and mutant “apo-form” were also performed using 2YFX PDB model as the 5A9U or 6MX8 PDBs were structurally similar (RMSD Cα deviation of 2YFX to 5A9U and 6MX8 was <0.29 Å). Molecular dynamics simulations were performed using the OpenMM engine(1). The Amber force field ff19SB(2) was used for protein, GAFF2 force field for the ligand(2), TIP3P model (3) was used for water and the system was neutralized using 150 mM NaCl with Amber tleap. Parameters for the MD equilibration protocol was that the energy minimization step performed in 5000 steps with the temperature and pressure set to 298 kelvin and 1 bar respectively. Position restraints force constant was set to 0 kJ/mol to allow backbone conformational flexibility to visualize the changes caused by the mutation in comparison to the WT. Equilibration MD simulation was performed and resulting coordinate file was used as the input file for the production MD simulation of 100 ns. The binding free energy was calculated using MM-PBSA(4).

**Data analysis**

Primary and secondary analysis of sequencing data (e.g. FASTQ-file generation, read alignment to the hg19 reference genome, VCF-file generation) were done using the onboard TruSight Oncology 500 v2.2 Local App (Illumina, San Diego, CA, USA). For variant classification, the small variants file (.genome.vcf), the copy number variants file (.vcf), the MSI file (.msi.json) and the TMB file (.tmb.json) from DNA sequencing along with the RNA fusions file (fusions.csv) from RNA-based sequencing were adapted to Molecular Health file format using MH VCF Adapter Suite version 2.1.0 and uploaded to the Molecular Health Guide database platform (version 5.3.0) (Molecular Health GmbH, Heidelberg, Germany). Furthermore, visual verification of called variants as well as RNA sequencing data was done using the open-source software integrative genome viewer version 2.16.1(5).

**Review of Litrature**

First, the databases COSMIC(6), OncoKB (prognostic & diagnostic levels)(7), ClinVar (clinically relevant variation)(8), cBioPortal(9), Cancer Genome Interpreter (CGI)(10), Drug-Gene-Interaction database (DGIdb)(11), CIVIC(12) and PubMed were applied in order to approve the recurrence of our genomic alteration in the *ALK*-gene. Second, ProteinPaint was exerted to check the functional relevance of the alteration(13).

**Inhibitors**

Crizotinib, brigatinib and lorlatinib were purchased from Selleckchem (Houston, USA). All the inhibitors were dissolved in dimethyl sulfoxide to make stock solutions of 10mM and stored at –20°C.

**Cell culture and DNA constructs**

Ba/F3 cells were obtained from the German Resource Centre for Biological Material (DSMZ) in 2023 and authenticated by DSMZ by DNA typing, species PCR and immunophenotyping. Cells were passaged for less than 6 months and were maintained in the presence of 2ng/ml interleukin-3 (R&D, Wiesbaden, Germany).

Ba/F3 cells were transfected by retroviral gene transfer and transformed upon withdrawal of interleukin-3. EML4/ALK cDNA is kindly gifted from Prof. Justus Duyster Lab
(University clinic, Freiburg, Germany). Wild-type EML4/ALK was cloned into PIG (MSCV-EGFP-PURO)-based retroviral vector by using EcoRI and Bam H1 restriction enzymes. The R1181H mutation was introduced in PIG- EML4/ALK using the QuickChange mutagenesis kit (Stratgene, Amsterdam, The Netherlands).

**Proliferation assay**

Proliferation was measured using an MTS (3-(4,5 dimethylthiazol-2-yl)-5-(3-carboxymethoxyphenyl-2-(4-sulfophenyl)-2H-tetrazolium)-based method by absorption of formazan at 490 nm (CellTiter 96; Promega, Madison, WI). Measures were taken as triplicates after 48 and 72 hours of culture without cytokines, as described previously(14). IC50 value is calculated using Prism Software.

**Western blot**

Ba/F3 cells were cultured for 3.5 hours without and in the presence of inhibitors crizotinib, brigatinib and lorlatinib at the indicated concentrations. Cell lysis, sodium dodecyl sulfate–polyacrylamide gel electrophoresis (SDS-PAGE), and immunoblotting were done as described previously(15). Phosphotyrosine antibodies were purchased from Upstate Biotechnology (4G10 and PY20) (Biozol, Eching, Germany). ALK, STAT5, pSTAT5, STAT3, pSTAT3, AKT, pAKT, ERK1/2, pERK1/2, JAK1 and pJAK1, JAK2, pJAK2, caspase-3, cleaved caspase-3, BCL-2, and beta-actin antibodies were obtained from cell signaling technology (Heidelberg, Germany). Bands were visualized using the enhanced chemiluminescence (ECL) system (Amersham, Braunschweig, Germany).

**Annexin staining**

Ba/F3 cells were cultured without and in the presence of inhibitors crizotinib, brigatinib and lorlatinib with the indicated concentrations for a period of 48hrs. After treatment with the ALK inhibitors, Ba/F3 cells are washed with 1x PBS and stained with annexin-FITC antibody (BD Biosciences, Heidelberg, Germany) for 20 minutes at 4 degrees. Before Fluorescence-Activated Cell Sorting (FACS) cells were washed again with 1X PBS and collected in FACS tubes. Annexin positive cells were analyzed with Flow Jo software.

**Statistics**

All statistical investigations were conducted using Graph-Pad PRISM 9 (San Diego, CA, USA), R-Studio v. 3.6.1 (Boston, MA, USA) and SPSS 26 (IBM, Armonk, NY, USA). Additionally, BioRender (Toronto, Canada) was used as a tool for visualization.

**Ethical statement**

Written informed consent for use of clinical data and as well as scientific use of biopsy material (including additional comprehensive genomic profiling) was obtained from the patient.

**Supplementary results from *in vitro* functional validation**

In order to identify if the EML4/ALK^R1181H^ variant drives resistance towards crizotinib, we cloned the non-mutated EML4/ALK and EML4/ALK^R1181H^ in a retroviral vector PIG (plasmid-IRES-EGFP-Puromycin). We established Ba/F3 cell with EML4/ALK and EML4/ALK^R1181H^ and measured the cell growth in the absence of IL-3. Both EML4/ALK and EML4/ALK^R1181H^ give similar levels of IL-3 independent growth compared to parental Ba/F3 cells indicating that the EML4/ALK^R1181H^ variant is oncogenic (**Figure 3a**; **Supplementary Figure 4**). Next, we sought to identify the crucial signaling pathway activation in EML4/ALK cells versus EML4/ALK^R1181H^ variant expressing cells and found that STAT3, ERK and AKT are strongly activated (**Figure 3b**) but not STAT5 and JAK1 (**Figure 3b**) suggesting that activation of STAT3 independent of JAK-family kinases, MAP-kinase and PI3K pathways are significantly activated in ALK mutated cells compared to mock transfected cells. Analysis of resistance towards the ALK inhibitors results clearly suggested that the EML4/ALK^R1181H^ variant shifted its cellular IC50 value (~300nM) three times more than the EML4/ALK (~105nM) towards crizotinib (**Figure 3c**). Biochemical analysis of inhibition of STAT3 and ALK phosphorylation results are also in line with cell proliferation data in the presence of crizotinib. These experiments show that STAT3 and ALK are inhibited at 100nM crizotinib concentration in EML4/ALK whereas EML4/ALK^R1181H^ displayed inhibition at 400nM crizotinib concentration (**Figure 3d**). Similarly, the EML4/ALK^R1181H^ variant also increased its cellular IC50 value (~55nM) compared to EML4/ALK (~25nM) towards brigatinib (**Figure 3e** and **Supplementary Table 3**). Consistent to the cell proliferation data, biochemical analysis of ALK and STAT3 phosphorylation data also suggest that 25nM concentration is able to inhibit the ALK and STAT3 activation in EML4/ALK cells whereas a 50nM concentration is required in order to inhibit the EML4/ALK^R1181H^ variant (**Figure 3f).** Next, we analyzed the role of lorlatinib, another ALK third generation inhibitor, and found that lorlatinib is very potent towards the EML4/ALK and EML4/ALK^R1181H^ (**Figure 3g**). Both STAT3 and ALK were inhibited at 0.25nM concentration in EML4/ALK cells and 1.25nM concentration was required to inhibit the EML4/ALK ^R1181H^ variant (**Figure 3h**). However, both variantare inhibited at nano molar concentration of lorlatinib and brigatinib compared to crizotinib suggesting that second generation ALK inhibitors are more potent towards EML4/ALK variants which drive partial resistance towards crizotinib. In addition, we also analyzed the induction of apoptosis after ALK inhibitor treatment in these cells and found that the EML4/ALK^R1181H^ variant is partially resistant towards crizotinib (**Figure 3i** and **Supplementary Figure 5a**) compared to brigatinib (**Figure 3j** and **Supplementary Figure 5b**) and lorlatinib (**Figure 3k** and **Supplementary Figure 5c**).

**Supplementary Tables**

| **Model** | **zDOPE score** |
| --- | --- |
| 2YFX | -1.86223 |
| 5A9U | -1.86455 |
| 6MX8 | -1.32 |

**Supplementary Table 1.** zDOPE score of edited PDB models using Modeller used as initial coordinates for simulations.

|  | Energy kcal/mol | Electrostatic | Van der Waals | Total |
| --- | --- | --- | --- | --- |
| 2YFX | WT | -8.22 ±3.11 | -42.68 ± 3.53 | -50.9 ± 3.74 |
|  | R1181H | -7.82 ± 3.56 | -41.03 ± 5.23 | -48.85 ± 3.2 |
|  |  |  |  |  |
| 5A9U | WT | -8.9 ± 1.6 | -43.62 ± 3 | -52.52 ± 2.93 |
|  | R1181H | -9.49 ± 1.8 | -45.55 ± 2.72 | -55.04 ± 2.79 |
|  |  |  |  |  |
| 6MX8 | WT | -9.65 ± 1.79 | -55.683 ± 3.28 | 65.333 ± 5.29 |
|  | R1181H | -8.86 ± 1.67 | -50.656 ± 3.02 | -59.516± 7.19 |

**Supplementary Table. 2.** Calculated binding energy of the ALK-ligand complex of the wildtype and the mutant.

| **Crizotinib** | **IC50 (nM)** |
| --- | --- |
| EML4/ALK | 105 |
| EML4/ALK^R1181H^ | 300 |
| **Brigatinib** | **IC50 (nM)** |
| EML4/ALK | 25 |
| EML4/ALK^R1181H^ | 55 |
| **Lorlatinib** | **IC50 (nM)** |
| EML4/ALK | 0.25 |
| EML4/ALK^R1181H^ | 1.25 |

**Supplementary Table 3.** IC50 values for both variants (EML4/ALK and EML4/ALK^R1181H^) in accordance to each ALK-inhibitor applied *in vitro*.

**Supplementary Table 4.** Data set of the first and second round (confirmatory round) of Western blots performed (separate Excel sheet).

**Supplementary Figures**

**
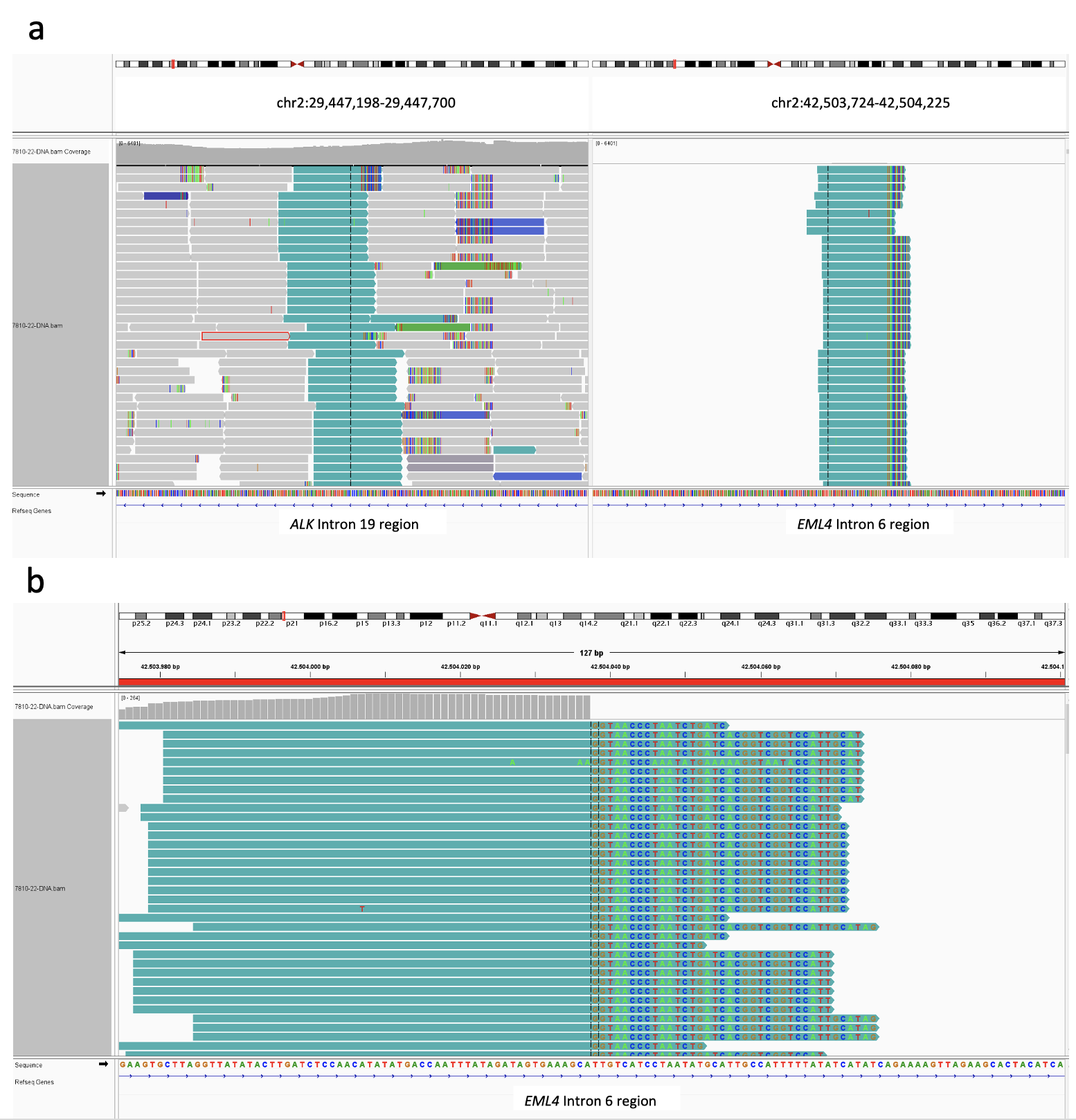
**

**Supplementary Figure 1.**  **(a)** Integrative Genome Viewer (IGV) display of DNA-sequencing reads. The alignment is sorted and colored by insert size and pair orientation. Shown is the “view mate in split screen” mode. The left panel shows reads aligning to the *ALK* gene intron 19 region. Teal bars represent reads with their respective mates starting in the *EML4* intron 6 region (shown in the right panel) identifying the *EML4-ALK* translocation. **(b)** Zoom into reads aligning to the *EML4* intron region 6*.* Teal bars represent matching reads whereas the colored letters mismatch but align to the *ALK* intron 19 region as revealed by a blastn search of the mismatched nucleotides. Additional RNA sequencing revealed genomic breakpoints at positions chr2:42492089 (*EML4*) and chr2:29446394 (*ALK*), data not shown.


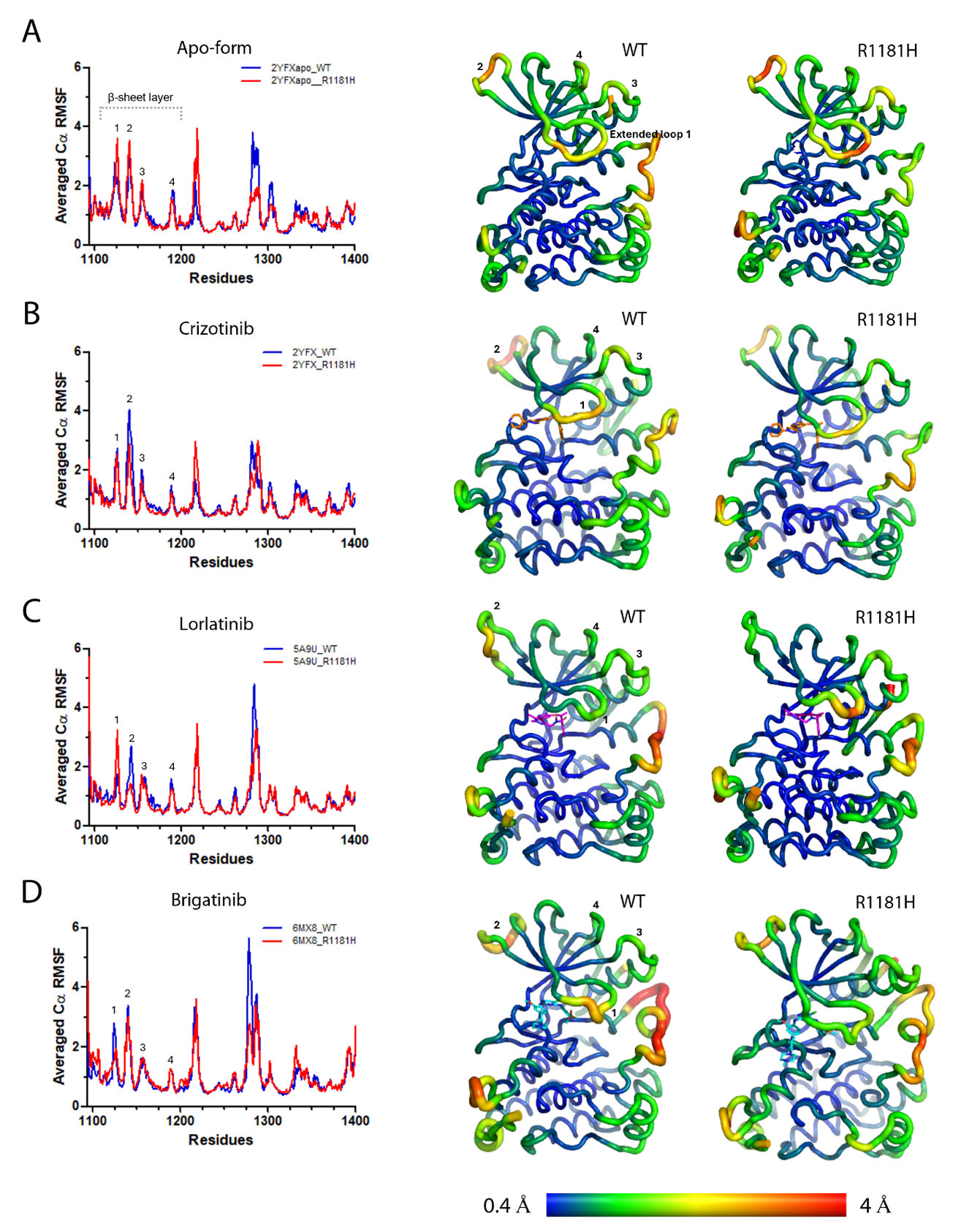


**Supplementary Figure 2.** Averaged Cα Root-mean squared fluctuations (Cα RMSF) of the wildtype and the mutant of the ligand complexes over 100 ns of MD simulations. A to D) The left inset shows the plot, Cα RMSF of the residues (wildtype in blue and mutant in red). The loops between the β-sheet layer are highlighted in the plot with notations 2 to 4 and extended loop 1 and also on the ligand-complex structures of the wildtype and the mutant respectively. The right inset depicts the Cα RMSF of each residue color-coded from blue to red indicating regions of low to high fluctuations during MD simulations (0.4 to 4 Å).

**Supplementary Figure 3.** **EML4/ALK ^R1181H^ variant transform Ba/F3 cells and activates STAT3, ERK and AKT.** Proliferation of parental Ba/F3 cells and Ba/F3 cells expressing EML4/ALK or EML4/ALK ^R1181H^ in the absence of IL-3 was quantified by the relative optical density (OD) after 24,48 and 72 hours using an MTS (3-(4,5-dimethylthiazol-2-yl)-2,5-diphenyltetrazolium bromide) -based assay.


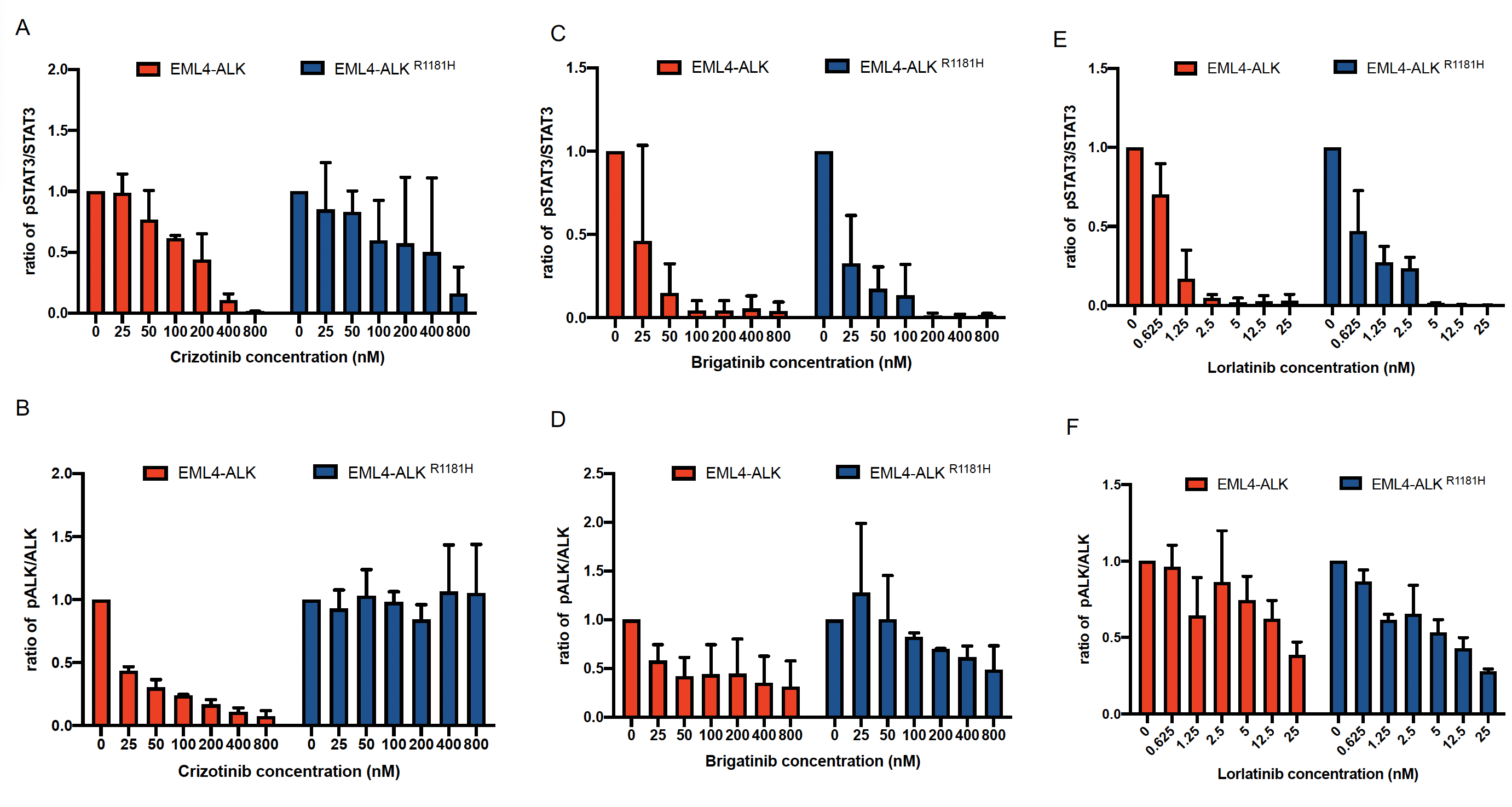


**Supplementary Figure 4.** Immunoblot analysis (n=2) of EML4-ALK and EML4-ALK ^R1181H^ variant expressing Ba/F3 cells were treated with indicated concentrations of crizotinib (0,25,50,100,200,400 and 800nM). (A) represents the ratio of pSTAT3/STAT3 with crizotinib treatment after normalized to untreated control. (B) represents the ratio of pALK/ALK with crizolitinib treatment. (C) and (D) represent the quantification data of the ratio of pSTAT3/STAT3 and pALK/ALK with brigatinib (0,25,50,100,200,400 and 800nM) respectively. (E) and (F) represent the quantification data of the ratio of pSTAT3/STAT3 and pALK/ALK with lorlatinib (0, 0.625,1.25,2.5,5, 12.5, and 25nM) respectively.

**
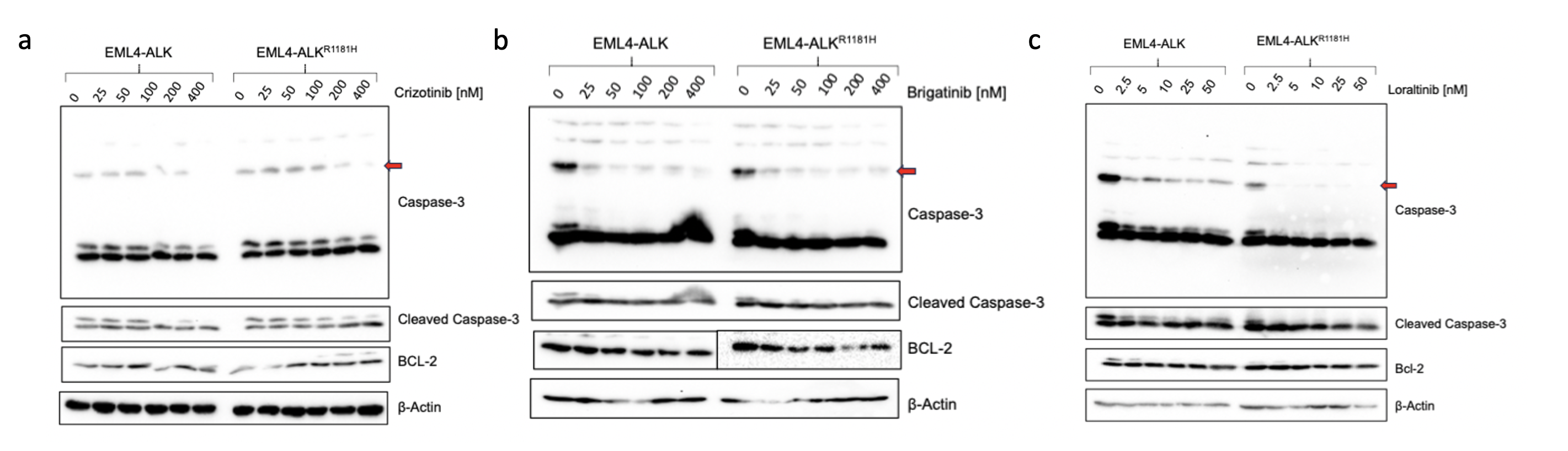
**

**Supplementary Figure 5.** **Brigatinib and lorlatinib are potent in the induction of apoptosis and EML4/ALK^R1181H^ variant confers only partial response towards crizotinib.** (a) Immunoblot analysis of Ba/F3 cells expressing EML4/ALK and EML4/ALK^R1181H^ cultured with indicated concentrations of crizotinib for 24hrs. A representative image of two independent experiments is shown (n=2). (b) Immunoblot analysis of Ba/F3 cells expressing EML4/ALK and EML4/ALK^R1181H^ cultured with indicated concentrations of brigatinib for 24hrs. A representative image of two independent experiments is shown (n=2). (c) Immunoblot analysis of Ba/F3 cells expressing EML4/ALK and EML4/ALK^R1181H^ cultured with indicated concentrations of lorlatinib for 24hrs. A representative image of two independent experiments is shown (n=2).

**References**

1. Arantes PR, Polêto MD, Pedebos C, Ligabue-Braun R. Making it Rain: Cloud-Based Molecular Simulations for Everyone. J Chem Inf Model. 2021;61(10):4852-6.

2. He X, Man VH, Yang W, Lee TS, Wang J. A fast and high-quality charge model for the next generation general AMBER force field. J Chem Phys. 2020;153(11):114502.

3. Tian C, Kasavajhala K, Belfon KAA, Raguette L, Huang H, Migues AN, et al. ff19SB: Amino-Acid-Specific Protein Backbone Parameters Trained against Quantum Mechanics Energy Surfaces in Solution. J Chem Theory Comput. 2020;16(1):528-52.

4. Genheden S, Ryde U. The MM/PBSA and MM/GBSA methods to estimate ligand-binding affinities. Expert Opin Drug Discov. 2015;10(5):449-61.

5. Robinson JT, Thorvaldsdottir H, Wenger AM, Zehir A, Mesirov JP. Variant Review with the Integrative Genomics Viewer. Cancer Res. 2017;77(21):e31-e4.

6. Forbes SA, Bhamra G, Bamford S, Dawson E, Kok C, Clements J, et al. The Catalogue of Somatic Mutations in Cancer (COSMIC). Curr Protoc Hum Genet. 2008;Chapter 10:Unit 10 1.

7. Chakravarty D, Gao J, Phillips SM, Kundra R, Zhang H, Wang J, et al. OncoKB: A Precision Oncology Knowledge Base. JCO Precis Oncol. 2017;2017.

8. Landrum MJ, Lee JM, Benson M, Brown GR, Chao C, Chitipiralla S, et al. ClinVar: improving access to variant interpretations and supporting evidence. Nucleic Acids Res. 2018;46(D1):D1062-D7.

9. Cerami E, Gao J, Dogrusoz U, Gross BE, Sumer SO, Aksoy BA, et al. The cBio cancer genomics portal: an open platform for exploring multidimensional cancer genomics data. Cancer Discov. 2012;2(5):401-4.

10. Tamborero D, Rubio-Perez C, Deu-Pons J, Schroeder MP, Vivancos A, Rovira A, et al. Cancer Genome Interpreter annotates the biological and clinical relevance of tumor alterations. Genome Med. 2018;10(1):25.

11. Freshour SL, Kiwala S, Cotto KC, Coffman AC, McMichael JF, Song JJ, et al. Integration of the Drug-Gene Interaction Database (DGIdb 4.0) with open crowdsource efforts. Nucleic Acids Res. 2021;49(D1):D1144-D51.

12. Griffith M, Spies NC, Krysiak K, McMichael JF, Coffman AC, Danos AM, et al. CIViC is a community knowledgebase for expert crowdsourcing the clinical interpretation of variants in cancer. Nat Genet. 2017;49(2):170-4.

13. Zhou X, Edmonson MN, Wilkinson MR, Patel A, Wu G, Liu Y, et al. Exploring genomic alteration in pediatric cancer using ProteinPaint. Nat Genet. 2016;48(1):4-6.

14. Gorantla SP, Dechow TN, Grundler R, Illert AL, Zum Buschenfelde CM, Kremer M, et al. Oncogenic JAK2V617F requires an intact SH2-like domain for constitutive activation and induction of a myeloproliferative disease in mice. Blood. 2010;116(22):4600-11.

15. Duyster J, Baskaran R, Wang JY. Src homology 2 domain as a specificity determinant in the c-Abl-mediated tyrosine phosphorylation of the RNA polymerase II carboxyl-terminal repeated domain. Proc Natl Acad Sci U S A. 1995;92(5):1555-9.
